# Conductive nerve conduit with piezoelectric properties for enhanced PC12 differentiation

**DOI:** 10.1038/s41598-023-38456-4

**Published:** 2023-07-25

**Authors:** Hamideh Javidi, Ahmad Ramazani Saadatabadi, S. K. Sadrnezhaad, Najmeh Najmoddin

**Affiliations:** 1 Department of Biomedical Engineering, Science and Research Branch, Islamic Azad University, Tehran, Iran; 2 Chemical and Petroleum Engineering Department, Sharif University of Technology, Tehran, Iran; 3 Department of Materials Science and Engineering, Sharif University of Technology, Tehran, Iran

**Keywords:** Biological techniques, Cell biology, Engineering, Materials science, Nanoscience and technology

## Abstract

Restoration of nerve tissue remains highly challenging, mainly due to the limited regeneration capacity of the nervous system and the development of fibrosis. This limitation necessitates designing new nerve guidance channel to promote nerve repairing. In this study, we developed a novel core/shell conduit to induce PC12 differentiation. Co-electrospinning method was utilized to produce a fibrous shell containing polycaprolactone/polyvinylidene fluoride PCL/PVDF, gelatin and polyaniline/graphene (PAG) nanocomposite. The core section of the conduit was filled with chitosan–gelatin hydrogel containing PAG and ZnO nanoparticles. Such conduit shows antibacterial activity, electrical conductivity and piezoelectric property. The effect of such engineered conduit on PC12 differentiation was investigated by analyzing differentiation markers Nestin and microtubule-associated protein 2 (MAP2) through immunocytochemistry and PCR-RT techniques. The result revealed that such conduit could significantly induce Nestin and MAP2 gene expression in the PC12 cells and, thus, it is a viable option for effective cell differentiation and nerve regeneration.

## Introduction

After injury, nervous system has an intrinsically limited ability to regenerate^1,2^. Tissue-engineered nerve guidance conduit (NGC) has emerged as a hopeful alternatives to grafts for repairing damaged nerve tissues^3,4^. To fabricate NGC with similar structure to extracellular membrane (ECM), the physiochemical, mechanical and biological properties of the materials such as biocompatibility, biodegradability, mechanical properties, minimum swelling and inflammation as well as desirable nerve conduction are important^4–6^. Beyond various reported techniques such as 3D printing^7^, gas foaming, freeze drying^5^, electrospinning is a cost-effective approach for production of fibrous and porous structures which could mimic the ECM^7–9^ and provide sufficient space for the growth and proliferation of cells^10^. Polycaprolactone (PCL) as a biocompatible polymer with semi-crystalline nature provides structural integrity and mechanical stability of the scaffold in tissue engineering^8,11^. Gelatin is a natural biopolymer which is widely exploited in the fabrication of scaffolds due to its high biodegradability, biocompatibility and great cell adhesion^12,13^. So, it can be used to improve the poor hydrophilicity and absence of cell attachment sites of PCL^8,11^. Chitosan (CS), derived from deacetylation of chitin has biocompatibility and antibacterial activity^14,15^ Considering the characteristics of gelatin, blending chitosan with gelatin compensates the lack of bioactivity of chitosan^16^.

Many research endeavors have revealed the effective role of the electrical force on cellular adhesion, proliferation, differentiation and migration of nerve cells^8,14^. Polyaniline (PANI) exhibits high chemical and thermal resistance as well as significant conductivity. Graphene with a single atomic sp^2^ sheet bonded carbon has received more attention in electrical conductivity^14^. It has been shown that the polyaniline/graphene (PAG) nanocomposite has a higher conductivity than PANI^8,17^ which could be beneficial for neural growth and differentiation^5,6,18^. PAG with highly electric conductivity and excellent chemical stability has been applied in the fabrication of conductive conduit. Boroojeni et al. incorporated PAG nanocomposite within gelatin nanofibers to endow the scaffold with conductive properties, which resemble the conductive behavior of axons^19^. Mohammadi et al. stated that multi-channel electrospun nerve conductive PCL/gelatin conduit containing 2% wt. PAG was the most favorable one for cell growth^8^. Soleimani et al. stated that PAG nanoparticles could improve cell adhesion and growth on a chitosan/gelatin-based scaffold^6^. Bayat et al. expressed the positive role of conductivity for cellular stimulation and growth by incorporation of PAG to the alginate guidance channel^5^.

Using a self-stimulated neural conductive channel to create a suitable scaffold for the growth and differentiation of neural-like cells can be desirable^20^. Exploiting piezoelectric species (e.g. polyvinylidene fluoride (PVDF), zinc oxide (ZnO)) as well-known self-electrical materials^21^ with the ability of inducing local electrical stimulation is a suitable alternative of implantable electrical stimulators or surface electrodes^20^.poly (vinylidene fluoride) (PVDF) have been a focal point of research due to its relatively intense piezoelectricity, thermostability, good mechanical properties, excellent biocompatibility and processing simplicity^22^. Due to the molecular chain of the β-phase which is responsible for the electrical feature in this material, in planar configuration the dipole moments are parallel which enhances the polar orientation under the external force compression^23,24^. Mohseni et al. revealed significant promotion of cell behavior in the self-electrical stimuli conductive gellan based scaffold containing short nanofibers of PVDF/MCM41 and 2% wt. PAG nanoparticles^23^. It has been reported that electrospun 20% wt. PVDF shows desirable electrical output^25^ and incorporation of ZnO nanostructure synergistically boosts the piezoelectric properties of PVDF^21^^,^^25^. Meanwhile, ZnO is a well-known antimicrobial and anti-inflammatory agent without side-effect on human cells. The liberation of Zn^2+^ from polymer matrix would be beneficial to prevent initial inflammation during the early stage of scaffold placement in the body^26^. PC12 cells are a useful in vitro model for neuronal differentiation. They respond to several growth factors, neurotrophins and hormones and can be utilized to assess distinct responses during differentiation^27^.

In this study, the aim was to investigate the effect of PAG nanocomposite particles on the differentiation of PC12 cell line and the expression level of nervous system genes nestin and MAP2. In this regard, the core/shell nerve conductive channel with a porous nanofiber structure filling with hydrogel has been designed for cell adhesion, and differentiation. The shell was made of co-electrospun PCL/PVDF along with gelatin fibers containing PAG nanoparticles and rolled in the form of channels. The core section of the conduit was filled with chitosan/gelatin hydrogels containing ZnO nanoparticles as well as PAG nanoparticles. One sample without PAG and one sample containing 2 wt. % PAG in the core and shell sections were fabricated and analyzed with various techniques. Finally the capability of fabricated conduits to differentiate PC12 cells was studied using real time-PCR and Immunocytochemistry.

## Materials and methods

### Materials

PCL pellets (M_n_ = 80,000 g mol^−1^), gelatin (porcine skin type A powder), polyvinylidene fluoride (PVDF; M_n_ = 270,000 g mol^−1^), aniline monomer, chitosan (Cs; deacetylation 75–85%), ammonium peroxydisulfate (APS), graphene, sodium dodecyl sulfate (SDS), fetal bovine serum (FBS), phosphate buffered saline (PBS), penicillin–streptomycin, and trypsin–EDTA, dey-engley neutralizing broth (DE), 4′,6-Diamidino-2-phenylindole dihydrochloride (DAPI), all were obtained from Sigma-Aldrich. The N, N-dimethylformamide (DMF), hydrochloric acid (HCl), glacial acetic acid, trypton soy agar (TSA), paraformaldehyde, Bovine serum albumin (BSA), ethanol, methanol, and acetone were purchased from Merck (Germany). Glutaraldehyde solution (GTA; 50%) was supplied by Beijing Chemical Reagents. Dulbecco′s modified DMEM/F12 was obtained from Gibco Invitrogen. SYBER Green Master Mix (Pluse 2x, High ROX™, Denmark), ZnO with a purity of over 99.5% was purchased from Bonyan Shimi Company, Tehran, Iran. Antibodies (MAP2, Nestin) and PC12 cells were obtained from Pasteur Institute, Tehran, Iran.

### Synthesis of polyaniline/graphene

The polyaniline/graphene (PAG) synthesis was performed according to Ref.^8,17^. To do this, a mixture of 5 g graphene in 100 ml HCl (1 M) containing aniline monomer was prepared through ultrasonic dispersion for 10 min to obtain foamy mixture. Then 5.767 g SDS was added to the vessel under nitrogen at room temperature, followed by the addition of 0.913 g APS and the stirring was continued for 6 h until the color was change from milky to bluish tint. After that, methanol was added to the mixture to stop the reaction. Next, washing step was repeated three times with ethanol, methanol, and distilled water. Finally, the synthesized PAG was dried in an oven at 50 °C for 48 h.

### Fabrication of electrospun scaffolds as a shell

13% w/v PCL solution in acetone (0.65 g in 5 ml) was obtained under magnetic stirrer at 50 °C for 2 h. Meanwhile, 0.95 g PVDF (19% w/v) was dissolved in 5 ml DMF via magnetic stirrer at room temperature for 1 h The PCL-PVDF solution was prepared by mixing the PCL and PVDF solutions (acetone to DMF ratio ~ 50:50) and the mixture was stirred for 2 h. A solution of 32% w/v (1.6 g in 5 ml) of gelatin was also prepared in acetic acid: water (60:40). The dispersed 2 wt. % PAG nanocomposite in distilled water was added to the gelatin solution and the mixing process was continued for 20 min.

The co-electrospinning process was carried out by 5 ml of each solution using 22_gauge needle during 8 h. The electrospinning parameters for each solution were as follows: Syringe 1 containing PCL/PVDF solution with a flow rate of 0.5 ml/h, applied voltage of 13 kV and tip to collector distance of 15 cm; and syringe 2 containing gelatin /PAG nanoparticles, the flow rate of 0.2 ml/h, the applied voltage of 10 kV and tip to collector distance of 16 cm. Two mats with and without PAG nanoparticles were electrospun and dried in a vacuum oven.

### Preparation of chitosan–gelatin hydrogel as a core

1% w/v of chitosan was dissolved in acetic acid, followed by 1 h incubation at 50 °C. Simultaneously, gelatin was dissolved in distilled water for 30 min at ambient temperature. Next, the gelatin solution and the chitosan solution were mixed by magnetic stirring for 30 min. Then, 1% w/v ZnO nanoparticles were dispersed in distilled water by ultrasonication for 2 min and added to the chitosan- gelatin solution. The dispersed 2 wt. % PAG nanocomposite in distilled water was poured to the chitosan- gelatin solution. Finally, hydrogels with and without 2% w/v PAG nanoparticles were placed in the freeze- dryer for 24 h.

### Preparation of conduits

**Figure 1:**
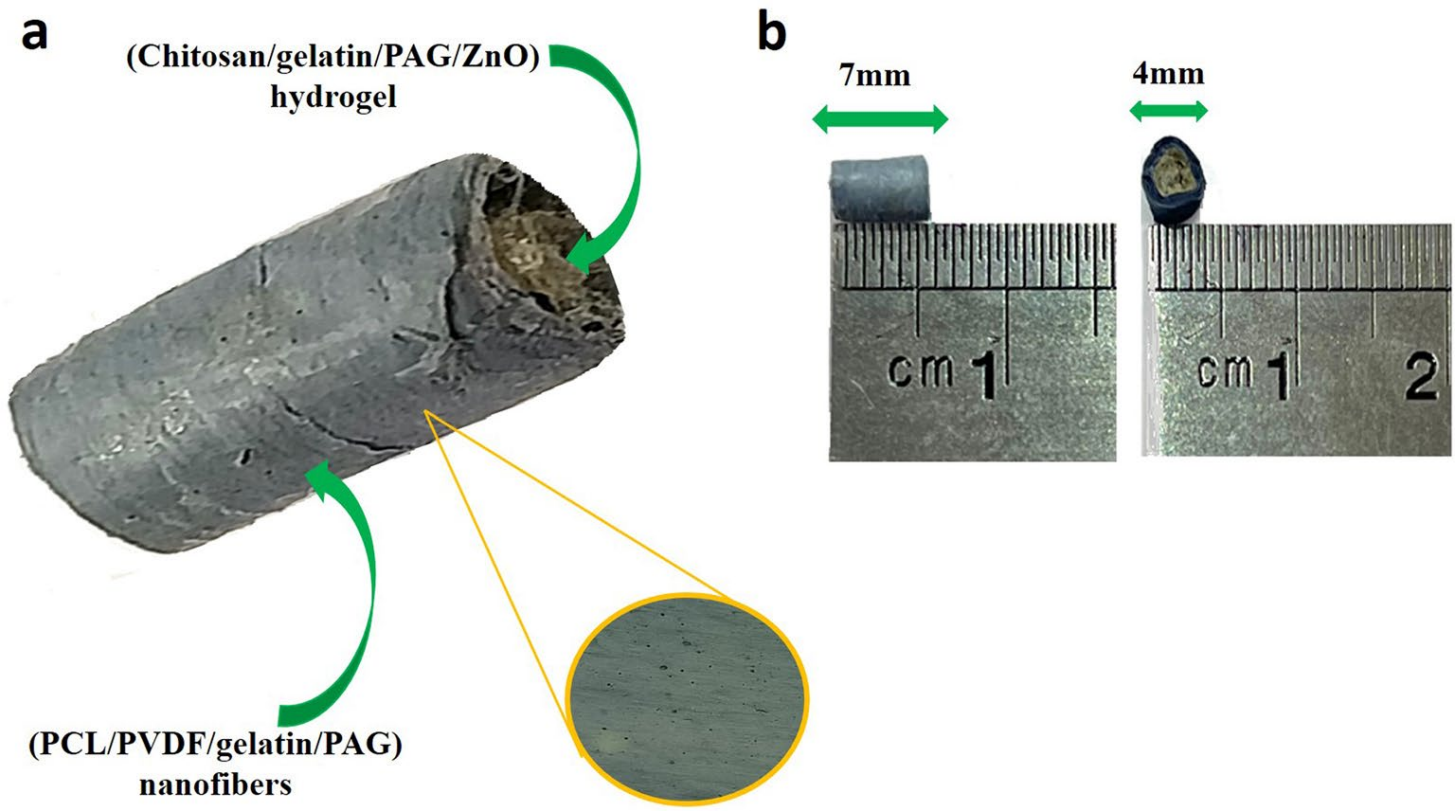
The photometric image of the conduit (**a**), the core–shell structure with its size (**b**).

Rectangular pieces of electrospun nanofibers scaffolds without and with 2 wt.% PAG nanoparticles were rolled around a Teflon tube and formed into a channel and were nominated as PAG0 and PAG2, respectively. The conduits were placed in the chamber of GTA steam (25 G v/v) for 48 h. After that, end of conduits was enclosed with an aluminum foil. PAG0 conduit was filled with a hydrogel containing Cs/gelatin/1 wt.% of ZnO and it was named as PCN0, whereas PAG2 conduit was filled with hydrogel containing Cs/gelatin/1 wt.% ZnO/2 wt.% of PAG and it was named as PCN2. Next, the top of both conduits were also covered and the conduits were placed in a freeze-drier for 24 h (Germany, CHRIST Alfa 1–2 LDplus). The photometric image of the conduit with core–shell structure as well as its shape and size is represented in Fig. 1.

### Characterization of conduit

The evaluation of textural features, microstructure of the conduit, cell morphology and attachment was performed through SEM imaging method using TESCAN (Mria3, Czech Republic) apparatus The functional groups were assessed by FTIR (Bruker, Ettlingen, Germany) within the range of 4000–500 cm^−1^. The wettability of the electrospun scaffold was evaluated using a contact angle meter (DSA25E, Kruss GmbH, Germany).

### Ethical approval

No human or animals used in this study.

#### Conduit degradation

To determine the in vitro conduit degradation rate, each sample was immersed in a 24-well plate containing PBS for 21 days. Then, the plate was placed in a shaker incubator with 60 rpm rotation at 37 °C. Finally, a filter paper was used to remove the remaining water and the in vitro degradation was calculated through Eq. (1).

1 $$D(\% ) = \frac{{W_{d} - W_{f} }}{{W_{d} }} \times 100\%$$

where *W*_*d*_ and *W*_*f*_ were the weight of cutting transverse of conduits before and after floating into PBS, respectively^8^.

#### Piezoelectric and conductivity measurements

To evaluate the piezoelectric property of nerve conduit, a mechanical moving arm was used. The output voltage was recorded by applying mechanical force to the sample and producing an electric field. The instrument applies a mechanical load approximately (1 ± 1 N) on 1 cm^2^ surface of the sample at a constant frequency of 1 Hz. Two Aluminum foils were attached to the sample and the electrical conductivity was measured by an oscilloscope.

The electrical conductivity of the samples was measured with two aluminum sheets attached to both sides of each sample by an oscilloscope, where the rate of current (I) was equivalent to 0.6 mA in Eq. (2).

2 $$\sigma = {\text{Ln}}^{{2}} /\pi {\text{ t }}\left( {{\text{I}}/{\text{V}}} \right)$$

where *σ*, *I*, *V*, and *t* represent electrical conductivity (S/cm), current (A), voltage (V), and thickness (cm), respectively^8,14^. The experiments were run in triplicate.

#### Cell attachment

For sterilization, the conduits were cut transversely and immersed in 70% ethanol for 60 min. After removing the scaffold from ethanol, the scaffolds underwent 15 min of PBS wash for three times. Both sides of the conduits were sterilized with ultraviolet radiation for 20 min. To investigate the adhesion capacity, PC12 cells were harvested in Dulbecco's Modified Eagle Medium/nutrient mixture F-12 (DMEM/F12, GIBCO Invitrogen) supplemented with 10% FBS and 1% antibiotic (penicillin–streptomycin) and were monitored for 7 days. For performing FESEM analysis, When cell confluency reached 80%, cells were seeded at a density of 1 $\times 10$^4^ cells/conduit and were fixed with 2.5% glutaraldehyde for 24 h. Each type of conduit was dehydrated with graded concentrations of ethanol (50, 60, 70, 80, 90, and 100% v/v) for 5 min and then dried under a fume hood^28^.

#### Anti-bacterial activity

The antibacterial activity of the fabricated PAG0 and PAG2 was expressed by half-McFarland standard^29^. This method is based on colony count unit (CFU), against both *Escherichia coli PTCC1399* (*E. Coli*) and *Staphylococcus aureus PTCC1112* (*S. aureus*) and as a Gram-negative and Gram-positive bacteria. Therefore, a half-McFarland suspension was made from bacteria. PAG0 and PAG2 nanofibrous scaffold were cut to 7 × 7 mm and an agar liquid was added to them, followed by incubation at 37 °C for 24 h. At the intended contact time, DE neutralizer was added to each sample at a dilution of 1:10 and the sample was sonicated for 1 min. Then, the suspension was cultured in TSA medium and the plates were incubated for 34 h. At the end, the number of viable bacterial colonies was counted. The following equation was used to evaluate the antibacterial activity.

3 $${\text{Antibacterial}}\,{\text{efficiency}}(\% ) = \frac{{{\text{CFU}}_{{{\text{control}}}} \, - {\text{ CFU}}_{{{\text{sample}}}} }}{{{\text{CFU}}_{{{\text{control}}}} }} \times 100\%$$

where *CFU*_*control*_ and *CFU*_*sample*_ are the average number of bacteria in the PAG0 and the PAG2, respectively.

#### Gene expression with polymerase chain reaction (real-time PCR)

Total RNA extraction from PC12 cells was performed using RNA isolation kit (Taiwan, Favorgen, Total RNA extraction mini kit). A cDNA synthesis kit (Thermo Scientific, USA) was utilized to convert total RNA into cDNA. Real-time PCR reactions were done using SYBR Green Master Mix (Amplicon RealQ Plus 2x, High ROX™, Denmark), For real-time PCR, all reactions were done under identical conditions, including 40 cycles of amplification with denaturation at 95 °C for 30 s, annealing at 60 °C for 20 s, and 30 s elongation at 72 °C for 40 cycles. Melting curve analysis and gel electrophoresis were used to determine the specificity of each primer set. The expression of target genes (Nestin and microtubule-associated protein 2 (MAP2)) was calculated after normalization with β-actin housekeeping gene and the 2^−∆∆CT^ method^28^. Specific primer sequences for each gene are summarized in Table 1.

**Table 1:** Primer sequences of neural differentiation genes.

Gene name	Forward primer	Reverse primer
β-actin	5-AGCACAGAGCCTCGCCTT-3	5-CACGATGGAGGGGAAGAC-3
Nestin	5-CTCCAGAAACTCAAGCACC-3	5-TCCTGATTCTCCTCTTCCA-3
MAP2	5-AGCTAGAGAGCCAGAGAGCC-3	5-CCCAATCAATGCTTCCTCGG-3

#### Immunocytochemistry

PC12 seeded conduits were analyzed after 21 days of incubation to express nestin and Map2 protein markers through ICC. The conduits were washed with PBS and fixed with paraformaldehyde %4 at 4º C with paraformaldehydehe cubation to expred twice with PBS. The immobilized cells were immersed in goat serum for 45 min. They were then immersed in Triton solution (4%) for 5 min. Primary nestin antibodies and MAP2 (Pasture Institute, Tehran, Iran) were incubated overnight at 4 °C and washed twice with PBS. Finally, 4,6-diamidino-2-phenylindole (DAPI, Sigma-Aldrich) was added to label the cell nuclei and incubated for 30 s. They were washed twice with PBS. Fluorescence microscopy (FV500, Olympus Fluoview, Japan) were utilized to obtain immunofluorescence images^30,31^.

## Statistical analysis

All data were analyzed by GraphPad Prism version 9.0.0, with the one-way ANOVA test. Data were expressed as the mean ± standard deviation (SD) of three experiments. *P* values lower that 0.05 were considered statistically significant.

## Results

### Characterization of conduit

**Figure 2:**
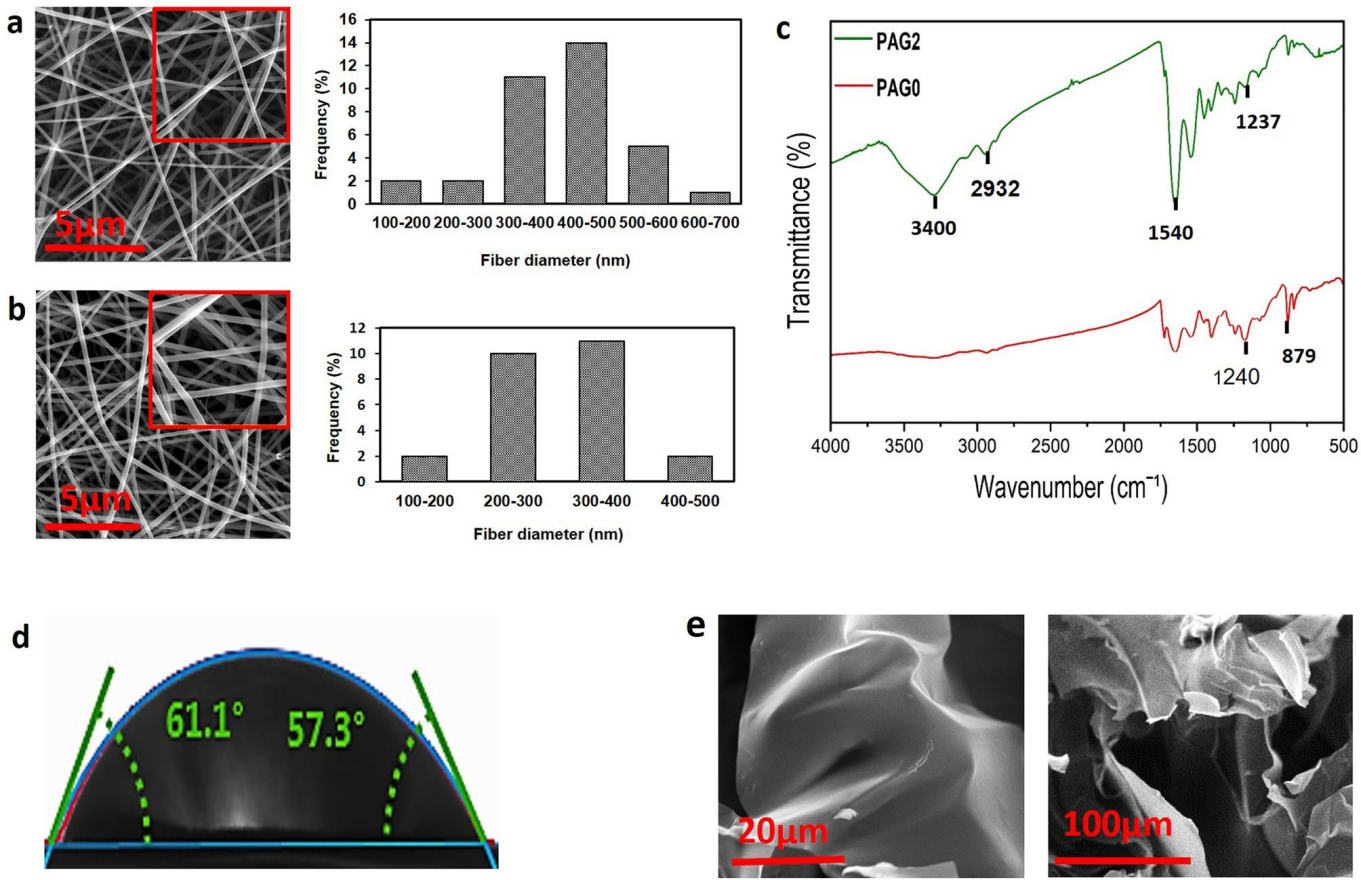
FESEM micrographs and the mean fibers diameter of PAG0 (**a**), PAG2 (**b**) nanofibrous shell. FTIR spectra of PAG0 and PAG2 fibrous mats (shell of conduits) (**c**); the average contact angle of PAG2 (shell of conduit) (**d**); FESEM micrographs of the CS–GEL/ZnO (1%)/PAG (2%) hydrogel (**e**).

To determine surface morphology of the proposed conduit, we used SEM imaging technique. The micrograph of PAG0 and PAG2 has been shown in Fig. 2a,b. As it can be seen, the prepared fibers had a smooth and uniform structure without any trace of beads. The right panel of the Fig. 1a,b shows the average diameter of nanofibers, according to which, the average diameter of PAG0 and PAG2 were 420 ± 112 nm and 198 ± 91 nm, respectively.

FTIR spectra of conduits shell (PAG0 and PAG2) is shown in Fig. 2c. The peak at 3400 cm^−1^ was attributed to N–H stretching. The bands in the range of 1500–1600 cm^−1^ related to the stretching of C–N of the benzenes and quinonics due to the presence of PAG nanocomposite in the shell of PAG2 conduit^4^. The peaks of PCL were observed at 1240 cm^−1^ (asymmetric C–O–C stretching) and at 2932 cm^−1^ (asymmetric CH_2_ stretching). The bands of gelatin were found to be at 3400 cm^−1^, 1540 cm^−1^, and 1240 cm^−1^ were assigned to the N–H stretching, C–N stretching and N–H, respectively. The bands related to the β-PVDF also were detected at 879 cm^−1^ and 1237 cm^−1^^32,33^ in both of conduits (PAG0 and PAG2). The contact angle of the conduit containing 2 wt.% PAG was around 55° ± 1° which is less than 90°, confirming the hydrophilic feature of fabricated conduit, is show in Fig. 2d.

SEM images of the CS/gelatin hydrogel containing PAG and ZnO is shown in Fig. 2e.

### Conduit degradation rate

**Figure 3:**
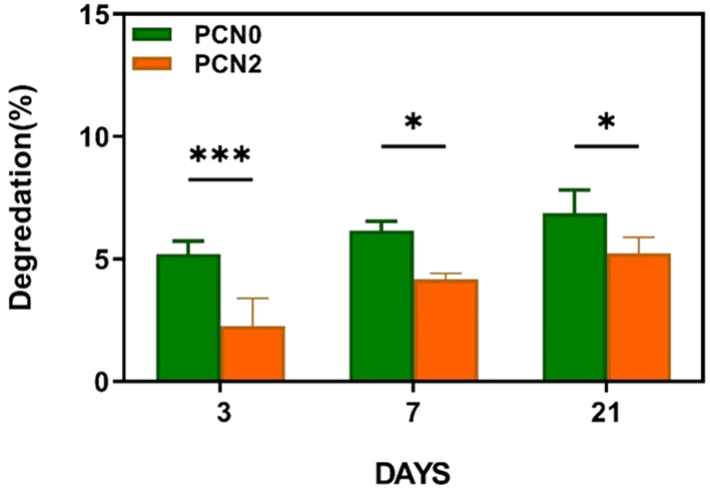
Degradation of the conduit without and with 2 wt.% PAG nanoparticles (PCN0, PCN2) after 3, 7 and 21 days. The obtained results are presented as the mean ± SD of at least three replicates. *p < 0.05, ***p < 0.001.

Figure 3 represents the in vitro degradation rate of fabricated conduits. It is evident that there is a significant difference between the biodegradation rate of PCN0 and PCN2. The lower degradation rate of PCN2 than that of PCN0 could be attributed to the presence of PAG nanocomposites in the core and shell sections of PCN2.

### Piezoelectric and conductivity analysis

Many research endeavors suggested that electrical stimuli play a crucial role in controlling cell function such as proliferation, differentiation and migration^5,11^. Using piezoelectric materials provides a good opportunity to induce localized electrical stimulation within the body in a non-invasive fashion^34^.

**Figure 4:**
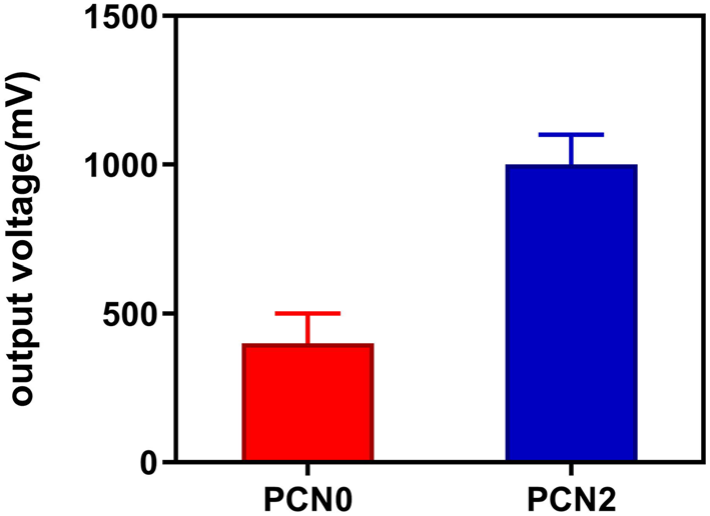
The effect of PAG nanocomposite on the output voltage of the conduit (PCN0 vs. PCN2).

Piezoelectric properties and electric conductivity of the PCN0 and PCN2 conduits have been reported in Fig. 4 and Table 2, respectively. The output voltage was found to be 400.3 ± 99 for PCN0 and 1000.3 ± 100 for PCN2. Moreover, there is a significant difference between electrical conductivity of PCN0 and PCN2. Incorporation of 2% PAG nanocomposite in to the conduit makes a significant increase in the output voltage and electrical conductivity.

**Table 2:** Electrical conductivity of the conduits without and with 2 wt.% PAG (PCN0 and PCN2) (*p* < 0.05).

Samples	PCN0	PCN2
Conductivity (S/cm)	(0.4 ± 0.20) × 10^–5^	(8.7 ± 0.62) × 10^–5^

### Cell proliferation and adhesion

**Figure 5:**
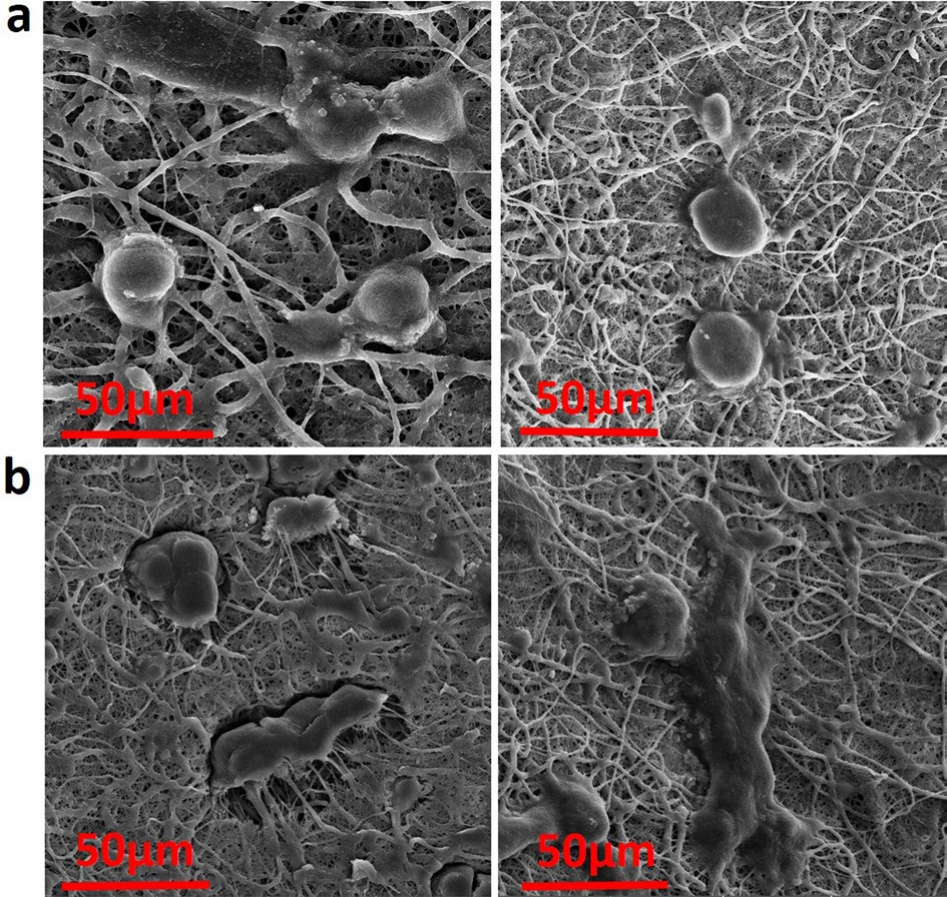
FESEM micrographs of PC12 cell adhesion and proliferation on the PCN0 (**a**) and the PCN2 (**b**) conduits.

The cell morphology and attachment of PC12 seeded on the fabricated PCN0 and PCN2 conduits were studied using SEM images after 21 days. Suitable attachment of cells is observed on both samples (Fig. 5). However, cell spreading on the surface of conduit is more pronounce for PCN2 than PCN0. Cells form long cytoplasmic branches and interact with pore walls across the scaffolds’ nanofibers. It has been stated that cells exhibit elongated shapes in desirable environments which could be a sign of biocompatibility of specific surfaces toward cell attachment^35^. It seems that the presence of 2 wt.% PAG nanocomposite in the neural guidance channel significantly contributed to the growth and adhesion of PC12 cells.

### Anti-bacterial activity

**Figure 6:**
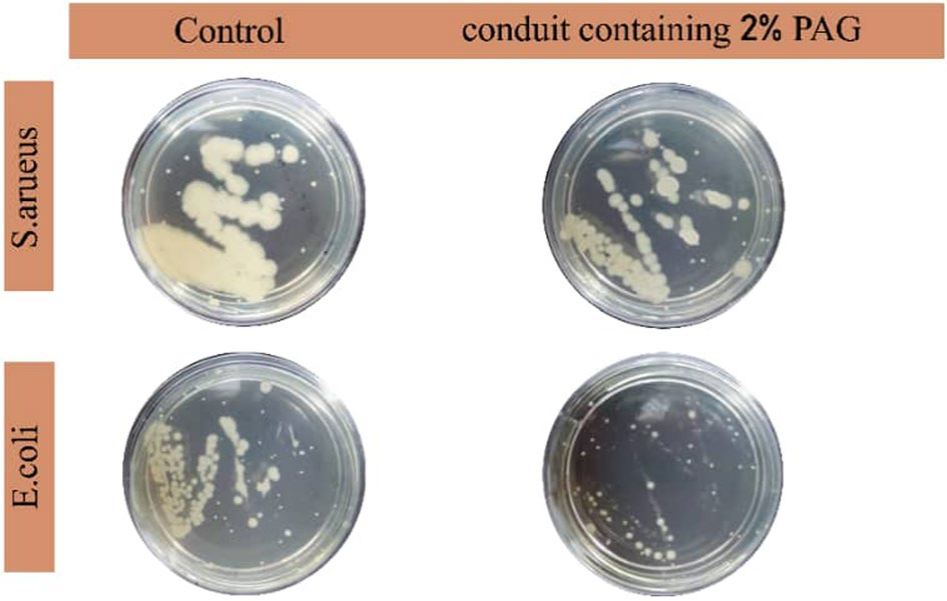
Anti-bacterial test of nanofibrous scaffold PAG0 and PAG2 without and with 2% PAG nanocomposit in shell structure.

The effect of PAG nanocomposite on the antibacterial property of fibrous scaffold was investigated using CFU method against both strains of Staphylococcus aureus and E. coli bacteria as shown in Fig. 6. The obtained results show that PAG2 inhibits growth by 25% and 51% on Staphylococcus aureus and E. coli bacteria strains, compered to PAG0. Graphene-based materials can interact with bacterial cells and prevent their growth due to their small size, functional groups, presence of free electrons and sharp edges of graphene nanosheets^11^. It is evident that such conduit has antibacterial properties which would be beneficial to be used in tissue engineering.

### PC12 differentiation

**Figure 7:**
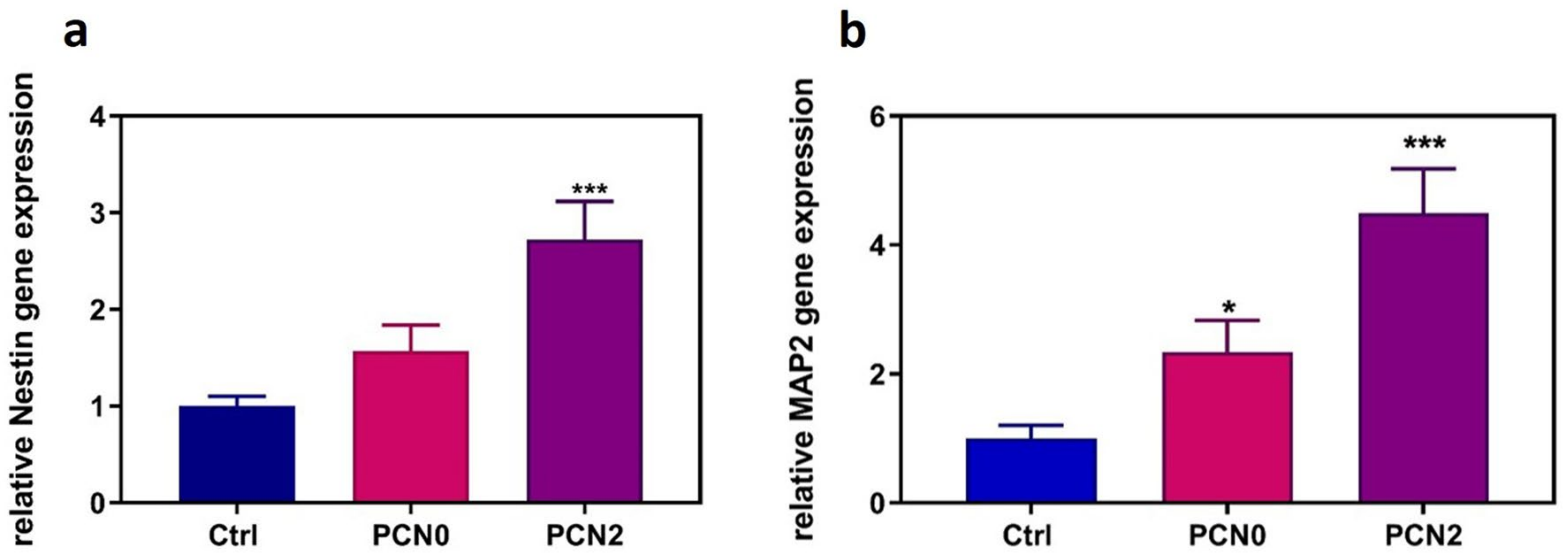
Nestin gene expression in the differentiated PC12 cells on PCN0 and PCN2 neural conduction channel (**a**); MAP2 gene expression in the differentiated PC12 cells on PCN0 and PCN2 neural conduction channel (**b**). The obtained results are presented as the mean ± SD of at least three replicates. *p < 0.05, **p < 0.01, ***p < 0.001.

In order to investigate the effect of PAG nanocomposite on cell differentiation, genes related to neural differentiation were analyzed through quantitative real-time PCR analysis and the immunocytochemistry (DAPI staining) was performed. Figure 7 represents Nestin and MAP2 gene expression under the effect of PCN0 and PCN2 conduits. The results of Nestin gene expression in cells adhered to both of conduit was significantly higher than control cells (*p* < 0.001). MAP2 gene expression was also found to be remarkably higher when cells adhered to PCN2 than PCN0. However, the effect of PCN2 on the MAP2 gene expression was more significant (*p* < 0.001).

**Figure 8:**
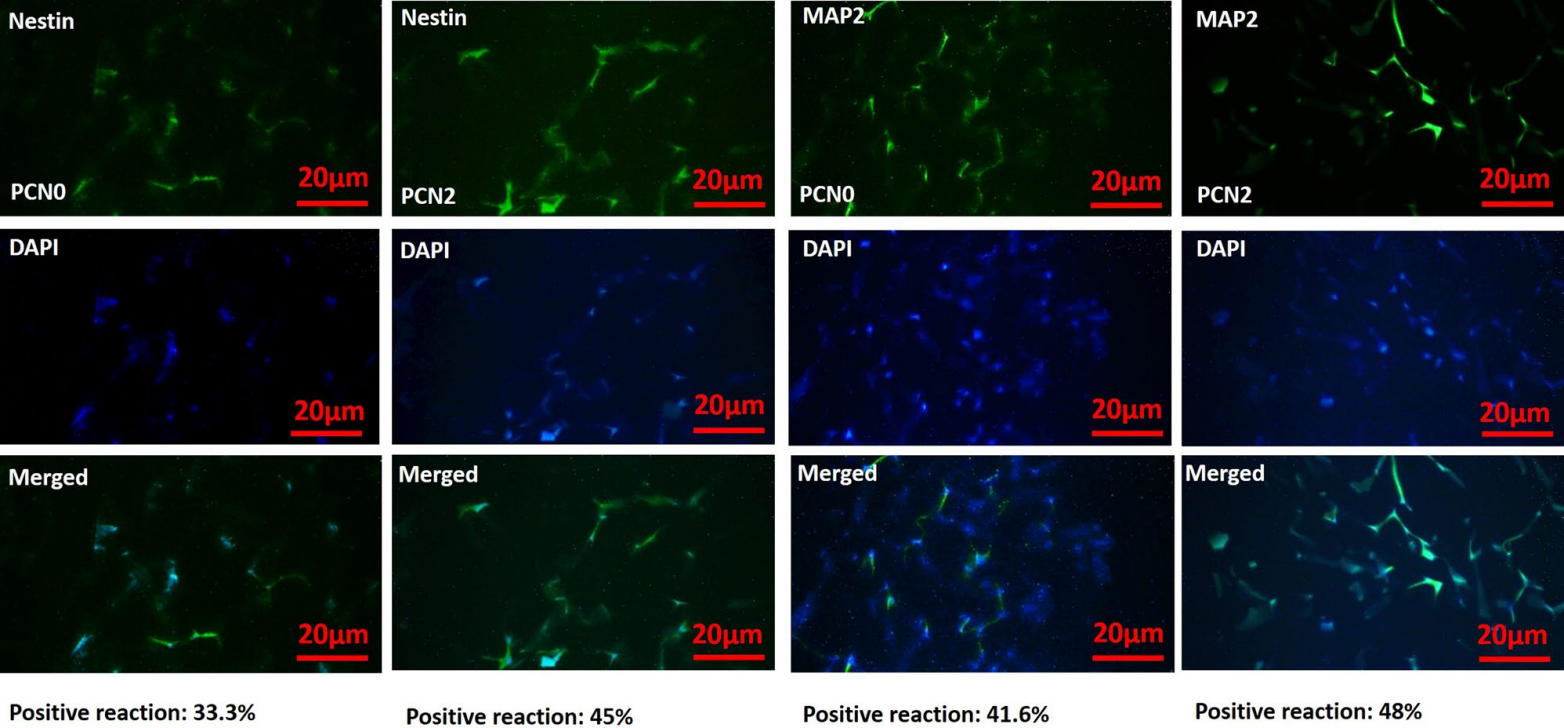
Immunostaining micrographs of PC12 cells cultured on the PCN0 conduit (neural guidance channel without PAG nanocomposite) and PCN2 conduit (neural guidance channel containing 2 wt.% PAG nanocomposite in core and shell structure).

The result of DAPI staining is shown in Fig. 8. It was found that PCN2 can efficiently induce higher levels of neurogenic proteins Nestin and MAP2 compared to PCN0. Furthermore, co-localization of DAPI showed PCN2 was found to have a higher reaction rate compared to PCN0. This could be ascribed to the presence of 2 wt.% PAG in the core and shell sections of the conduit which promotes electrical as well as piezoelectric properties of the composite.

## Discussion

Damaged nerves in the nervous system have a limited capacity for spontaneous regeneration^1,2^. This often leads to permanent disabilities, and thus it is important to regenerate injured nerves to recover the function of nervous system. Weak intrinsic regeneration capacity of nervous system entails the establishment of novel approach that can induce nerve repair and regeneration. Tissue engineering has provided cell-based methods to reduce therapeutic limitations^36^. Trying to fabricate a biodegradable and biocompatible scaffold with desirable porosity form natural and synthetic polymers for the growth, proliferation and differentiation of cells is very promising. The NGC provides a stable and elongated tool with similar structure of ECM to support the growth of axons for the purpose of establishing synaptic connections^37^. This study set out to investigate the effect of PAG nanocomposites on the PC12 differentiation and the ensuing nerve renewal. In this regard, we synthesized a novel core/shell structure that showed intriguing properties as a nerve conduit. PCL, PVDF, gelatin and PAG were the major components of the channel fibrous shell. Meanwhile, chitosan and gelatin formed the hydrogel and incorporated with PAG and ZnO nanoparticles as a core section of the conduit. PCL is biocompatible and biodegradable and providing integrity and mechanical properties of the shell^8,30^. However, its poor biological properties and hydrophobicity can be compensated using a natural polymer such as gelatin^8^. Chitosan (Cs) is a natural, biocompatible and biodegradable substance with antibacterial properties^14,16^. However, the bioactivity of CS is weak and hence biologically active materials like gelatin are blended^8^. PVDF as a well-known piezoelectric organic platform is lightweight, biocompatible, flexible and electroactive^38^. ZnO is a piezoelectric material with the ability of generating electricity in response to external mechanical stimulation. Additionally, it has an extended application in biomedical setting due to its ecofriendly, nontoxic, antibacterial and proangiogenic properties^21^. The use of PVDF in the fibrous shell and addition of ZnO nanoparticles in to the hydrogel provides piezoelectric properties in the shell and core structure of the conduit. Many researchers have shown that the electrical conductivity of PAG has a significant effect on the adhesion, growth, migration, and differentiation of nerve cells^6,23^.

SEM images showed that the incorporation of PAG reduced the fiber diameter of the fibers. This may be the outcome of increased electrical conductivity. The presence of PAG nanocomposite could also positively affect the preparation of smooth electrospun fibers. Moreover, addition of PAG could lead to decrease the biodegradability of the conduit. More exactly, PAG nanocomposite were capable of preventing premature degradation of the conduit, which can effectively enhance cell growth and survival on the conduit^8^. The antibacterial test revealed that the fabricated conduit had a potency of reducing the colony of *S. aureus* and *E. coli* bacteria. It was revealed that the PCN2 has a promising electric conductivity as well as piezoelectric property, leading to improve nerve guide function of the conduit. The most interesting result of this study was the increased gene and protein levels of specific neural-related markers such as Nestin and MAP2 under the effect of PAG nanocomposite. The active expression of Nestin and MAP2 during neural differentiation process has been reported in previous studies^39–41^. Nestin, which is the common name of neuroepithelial stem cell protein, is an intermediate filament that is deemed to be highly related to pivotal functions of stem cells including self-renewal, differentiation, proliferation and migration^39^. Notably, Nestin gene expression is transient and is mainly provoked during tissue development or pathological conditions to enhance tissue regeneration^42^. MAP2 is abundantly localized in the cell body and dendrites of neurons and has major contributions in the neural outgrowth and synaptic plasticity^43^. An important part of the neurogenesis process is microtubule assembly, in which MAP2 plays a critical role in neurite formation, which can further develop into dendrites and axons^42^. MAP2 has been found in the mature neural cell that is indicative of low MAP2 gene expression during early stages of differentiation^42^. Thus, increased expression and protein level of MAP2, which was observed in this study, shows that the PAG2 nanocomposite was able to promote neuronal differentiation and maturation of the conduit. It is interesting to note that gene expression level of both Nestin and MAP2, as well-known markers of neuronal differentiation, is significantly downregulated after the process of neuronal differentiation and maturation^42^. This means that these genes are activated in the presence of a stimulus of differentiation and herein the administration of the conductive conduit could effectively recruit both Nestin and MAP2 markers, and consequently induce PC12 cell differentiation. The significant effect of PAG nanocomposite in the nerve conduction conduit on the growth, adhesion and differentiation of PC12 cells is evidence of the extraordinary ability of PAG nanocomposites on nerve conduction and can be a favorable option for the repair and renewal of the nervous system in tissue engineering.

## Conclusion

Returning to the hypothesis posed at the beginning of this study, it is now possible to state that the core/shell structure of PCN2 conduit with electrical conductivity as well as piezoelectric property has a high potency for nerve repair and regeneration. The most obvious finding to emerge from this study is that the PCN2 conduit has the ability to induce cell differentiation and nerve reconstruction. This study has gone some way towards our understanding about the role and contribution of conduit containing PAG in nerve regeneration process. However, more research is needed to better understand the function and clinical outcome of the PCN2 conduit.

## Data Availability

The datasets used and/or analyzed during the current study are available from the corresponding author on reasonable request.
